# Effect of Short-Term Tacrolimus Exposure on Rat Liver: An Insight into Serum Antioxidant Status, Liver Lipid Peroxidation, and Inflammation

**DOI:** 10.1155/2021/6613786

**Published:** 2021-02-24

**Authors:** N. Fatima, N. Sheikh, A. R. Satoskar, T. Akhtar, A. Tayyeb, I. Ashfaq, N. Ryan, S. Ambreen, B. K. Jha, S. Oghumu

**Affiliations:** ^1^Cell and Molecular Biology Lab, Institute of Zoology, University of the Punjab, Q-A Campus, Lahore 54590, Pakistan; ^2^Department of Pathology and Microbiology, Wexner Medical Center, The Ohio State University, Columbus, OH 43210, USA; ^3^Department of Pharmacology, University of Health Sciences, Lahore 54600, Pakistan; ^4^School of Biological Sciences, University of the Punjab, Q-A Campus, Lahore 54590, Pakistan; ^5^Institute of Laboratory Medicine, Clinical Chemistry and Molecular Diagnostics, University of Leipzig, Germany

## Abstract

Tacrolimus (TAC) is an immunosuppressive drug, optimally used for liver, kidney, and heart transplant to avoid immune rejection. In retrospect, a multitude of studies have reported effects of TAC, such as nephrotoxicity, diabetes, and other complications. However, limited information is available regarding short-term exposure of TAC on the liver. Therefore, the present study was designed to unravel the effects of short-term exposure of TAC on a rat model. The animal model was established by TAC administration for 6, 12, 24, and 48 h time points. Liver histopathological changes were observed with PAS-D, reticulin stain, and immunostaining of PCNA and CK-7 coupled with glycogen quantification in a liver homogenate. TUNEL assay was performed to evaluate the DNA damage in the liver. Concentration of GSH and activities of SOD and CAT in the serum were measured to assess the antioxidant status, whereas liver tissue MDA level was measured as a biomarker of oxidative stress. Hepatic gene expression analysis of IL-10, IL-13, SOCS-2, and SOCS-3 was performed by RT-PCR. Results revealed marked changes in liver architecture of all TAC-treated groups, as evidenced by sinusoid dilation, hepatocyte derangement, glycogen deposition, and collapsed reticulin fibers. Significant increase in PCNA and CK-7 immunostaining along with the presence of TUNEL-positive cells was revealed in treatment groups as compared to the control group. Serum antioxidant enzyme status was markedly decreased, whereas the liver MDA level was increased in TAC treatment groups indicating oxidative stress induction. The gene expression profile of cytokines was significantly upregulated in treatment groups highlighting an inflammatory response. In conclusion, results of the current study propose that even a short-term TAC exposure can induce change in antioxidant status and lipid peroxidation. Therefore, these factors should be considered to avoid and minimize immunosuppression-related issues in a prolonged course of treatment.

## 1. Introduction

Tacrolimus (TAC) is one of the widely used immunosuppressive drugs (ISD) for the prophylaxis of transplant patients [1]. TAC belongs to the category of calcineurin inhibitor (CNI); these drugs work by inhibiting calcium-dependent events and are being used for liver transplantation since 1998 [2, 3]. Although use of TAC has markedly reduced the risk of rejection, adverse effects of immunosuppressive drugs are still a major concern [4]. TAC works by binding to an immunophilin FK506 binding protein (FKBP) and after complex formation, inhibits calcium-dependent events, as a result T cell proliferation is inhibited which impairs T cell-mediated cytotoxicity. Furthermore, B cell growth and antibody production is also affected due to suppression of T cell-derived growth factors, and antigen presentation is spared [5]. Myriads of adverse effects and contraindications of TAC have been recently reported in transplant patients which include hepatotoxicity, encephalopathy, diabetes mellitus, nephropathy, increased susceptibility to COVID-19, and other infectious diseases, owing to immunosuppression [6–10]. Previous studies have reported nephrotoxicity in patients receiving TAC as immunosuppressive therapy after liver transplant. TAC-induced renal and pulmonary toxicity has been reported with morphopathological alterations and cellular necrosis [11, 12]. Previous studies have also reported development of hepatic infarction in post liver transplant patients, as an effect of TAC [13]. Immunosuppressive drugs have some off target effects which may lead to production of reactive oxygen species (ROS) and induction of apoptotic cell death due to impaired mitochondrial and T cell functions [14]. Therefore, liver-targeted management should be taken into account to avoid liver complications [15, 16].

Association of TAC and change in oxidative stress marker 8,12-iso-isoprostane F-2alpha-VI has been described previously in heart transplant patients [17]. Oxidative stress and mitochondrial dysfunction has also been reported in an in vitro organoid model of nephrotoxicity in TAC-induced renal injury characterized by oxidative stress and production of inflammatory cytokines [9, 18]. Furthermore, DNA damage and expression of proliferating cell nuclear antigen (PCNA) and cytokeratin 7 (CK-7) are important biomarkers to assess liver injury. PCNA and CK-7 immunohistochemistry and gene expression analysis have shown increased expression in hepatotoxicity [19–22]. Likewise, the cytokine profile is altered following liver injury. Proinflammatory cytokines, interleukin- (IL-) 10, and IL-13 exhibit an increased expression in rat liver injury to protect the tissue from damage [23]. A role of IL-10 has been previously described owing to progression of liver inflammation and oxidative stress-related liver injury. An upregulated expression of IL-10 and decreased activity of superoxide dismutase (SOD) with increased malondialdehyde (MDA) level has been reported due to cyclophosphamide-induced immunosuppression in rats [24]. These cytokines trigger activation of suppressor of cytokine signaling (SOCS) genes, which in turn can activate other signal transduction pathways, such as the JACK/STAT pathway, to promote cell proliferation and apoptosis [25].

Paucity of information regarding the oxidative effects of TAC on rat liver demanded a study to be designed with an aim to assess liver histopathological changes, DNA damage, expression of PCNA and CK-7, serum antioxidant status, lipid peroxidation level in the liver, and change in hepatic gene expression pattern of cytokines due to short-term exposure of TAC on rat.

## 2. Materials and Methods

### 2.1. Experimental Design

Male Wistar rats (250 ± 25 g) of 10 weeks old (sexually mature) were raised and caged in the animal house of the Institute of Zoology, University of the Punjab, Lahore, Pakistan, after ethical committee approval (D/622/U.Z) and fed with normal rat chow and water *ad libitum*. After being acclimatized, rats were randomly divided into 4 experimental groups and one control group, having 9 animals in each group (*n* = 9). An oral dose of 3 mg/kg of TAC (Sigma, Cat. No. Y0001926) was administered once, for 6, 12, 24, and 48 h, as described previously [26]. The rats were sacrificed after the aforementioned time points with blood sample and liver tissue collection.

### 2.2. Histopathological Analysis

For histological analysis, a portion from the left lobe of the liver tissue was used, followed by fixation in formalin and embedding in paraffin for histological analysis. Paraffin-embedded tissue sections were cut 6 *μ*m thick, and slides with tissue sections were incubated overnight at 37°C. PAS-D, reticulin, immunostaining of PCNA and CK-7, and terminal deoxynucleotidyl transferase dUTP nick end labeling (TUNEL) staining were performed as a preliminary finding to detect liver injury.

### 2.3. PAS-D Staining

Formalin-fixed and paraffin-embedded (FFPE) tissue slides were subjected to PAS-D staining using a kit (Sigma-Aldrich Periodic Acid-Schiff (PAS) Staining System 395) according to the manufacturer's protocol, as described previously [27].

### 2.4. Reticulin Staining

Reticulin staining was performed using a Reticulum Stain kit (Sigma-Aldrich, HT102) according to the manufacturer's protocol. Briefly, tissues were deparaffinized, hydrated, and oxidized in potassium permanganate solution with a quick rinse in tap water. Slides were subjected to bleaching agent, oxalic acid followed by iron alum-treated sensitization for effective impregnation of silver. Sensitized slides were further exposed to an ammoniacal solution of silver nitrate solution. Reduction of tissue sections was carried out with aqueous formalin and toned with gold chloride solution, followed by removal of unreduced silver with sodium thiosulphate solution. Sections were then dehydrated, cleared, mounted, and observed using a light microscope.

### 2.5. Immunohistochemical Analysis

Immunohistochemistry was performed to identify the presence and extent of PCNA and CK-7 expression in liver tissue. Tissue sections were deparaffinized in xylene and rehydrated to water through graded ethanol. Heat-induced epitope retrieval was performed, and sections were washed by immersion in PBS. After washing, sections were blocked followed by another wash in PBS, and samples were incubated with 10% normal goat serum (Vector Laboratories S-1000) in a humidified chamber for 30 minutes, followed by incubation with primary antibody solution (PCNA MAB424, Millipore Sigma, USA, CK-7 RCK105) in 10% normal goat serum. Concentration of primary antibodies used was 1 : 100, and slides were incubated overnight at 4°C in a humidified chamber. After flicking off the primary antibody, slides were washed in PBS and incubated with biotinylated secondary antibody for 30 minutes (Vector Laboratories BP-9200) at room temperature (RT) in a humidified chamber. Tissue section without a primary antibody was kept as a negative control to check the background staining of liver tissue. After washing with PBS, slides were incubated with Streptavidin-HRP (SA-5004 Vector Laboratories) for 30 minutes at RT in a humidified chamber, followed by another PBS wash. Slides were finally incubated with DAB solution (Vector DAB substrate SK-4100) and washed with dH_2_O. Slides were further counterstained with Hematoxylin 2 (Fisher Scientific), dehydrated through graded ethanol to xylenes, mounted, and observed under a light microscope.

### 2.6. Assessment of DNA Damage by TUNEL Assay

DNA damage in all experimental groups was analysed using TUNEL assay (ab66108) according to the instructions provided, as described previously [27]. Three stained slides were observed, and the image was captured using a fluorescent microscope to observe the TUNEL-positive cells, and ImageJ software was used to count the cells.

### 2.7. Glycogen in Liver Tissue Homogenate

Liver tissue homogenate was used to determine the concentration of glycogen to further confirm the deposition of glycogen in liver tissue, as described previously [28].

### 2.8. Serum Antioxidants

#### 2.8.1. Glutathione Concentration

Serum glutathione (GSH) concentration was measured using a glutathione colorimetric detection kit (Invitrogen EIAGSHC). Briefly, serum was treated with 5% SSA and incubated for 10 minutes at 4°C followed by centrifugation at 14,000 rpm for 10 minutes at 4°C. The supernatant was collected and added in a 96-well plate followed by dilution with an assay buffer. Absorbance was read at 405 nm in a microplate reader.

#### 2.8.2. Superoxide Dismutase Activity

Serum samples were used to measure the activity of SOD using a SOD assay kit-WST (Sigma 19160), according to the manufacturer's instructions. Briefly, serum sample, dH_2_O, and WST working solution and enzyme working solution dilution buffer were added in a 96-well microplate, and the plate was incubated at 37°C for 20 minutes. Absorbance at 450 nm was read in a microplate reader.

#### 2.8.3. Catalase Activity

Activity of catalase (CAT) in serum samples was measured by a catalase activity colorimetric assay kit (ab83464) according to the manufacturer's instructions. Briefly, serum samples in a 96-well plate were treated with hydrogen peroxide and incubated for 30 minutes followed by addition of stop solution and development solution. Absorbance was read at 570 nm in a microplate reader.

### 2.9. Lipid Peroxidation Assay in the Liver

#### 2.9.1. MDA Level

Liver tissue homogenate was used to assess lipid peroxidation by measuring the level of MDA using the calorimetric assay kit (MAK085) according to the manufacturer's instructions. Briefly, tissue was lysed in MDA lysis buffer and proteins were precipitated. A sample was taken in a 96-well plate and treated with thiobarbituric acid, incubated at 95°C for 60 minutes followed by cooling at room temperature for 10 minutes. Absorbance was read at 532 nm in a microplate reader.

### 2.10. Gene Expression Analysis with Real-Time PCR (RT-PCR)

Expression profile of cytokines was determined by RT-PCR analysis. Freshly excised liver tissues were used for RNA extraction using the TRIzol method. After measuring the concentration of RNA with NanoDrop, cDNA was synthesized using a cDNA synthesis kit (Thermo Scientific) according to the manufacturer's protocol. Gene expression level of IL-10 (forward: TGTGAAAATAAGAGCAAGGCAGTG, reverse: CATTCATGGCCTTGTAGACACC), IL-13 (forward: CAGCATGGTATGGAGTGTGG, reverse: TGGGCTACTTCGATTTTGGT), SOCS-2 (forward: TCAGCTGGACCGACTAACCT, reverse: TGTCCGTTTATCCTTGCACA), and SOCS-3 (forward: AGCTCCAAAAGCGAGTACCA, reverse: TGACGCTCAACGTGAAGAAG) was investigated by a PikoReal™ Real-Time PCR system using a SYBR-GREEN master mix. Ct value was measured by an amplification curve, and results were normalized using GAPDH (forward: GAAACCTGCCAAGTATGA, reverse: GCTGTAGCCGTATTCATT) as an endogenous control.

### 2.11. Statistical Analysis

Statistical analysis was performed by one-way ANOVA, and data was normalized by Tukey's post hoc test using GraphPad Prism 5 (San Diego, CA). Results are presented as mean ± SEM, and *P* > 0.05 was considered as statistically significant.

## 3. Results

### 3.1. Short-Term Tacrolimus Exposure Induces Acute Liver Injury

**Figure 1:**
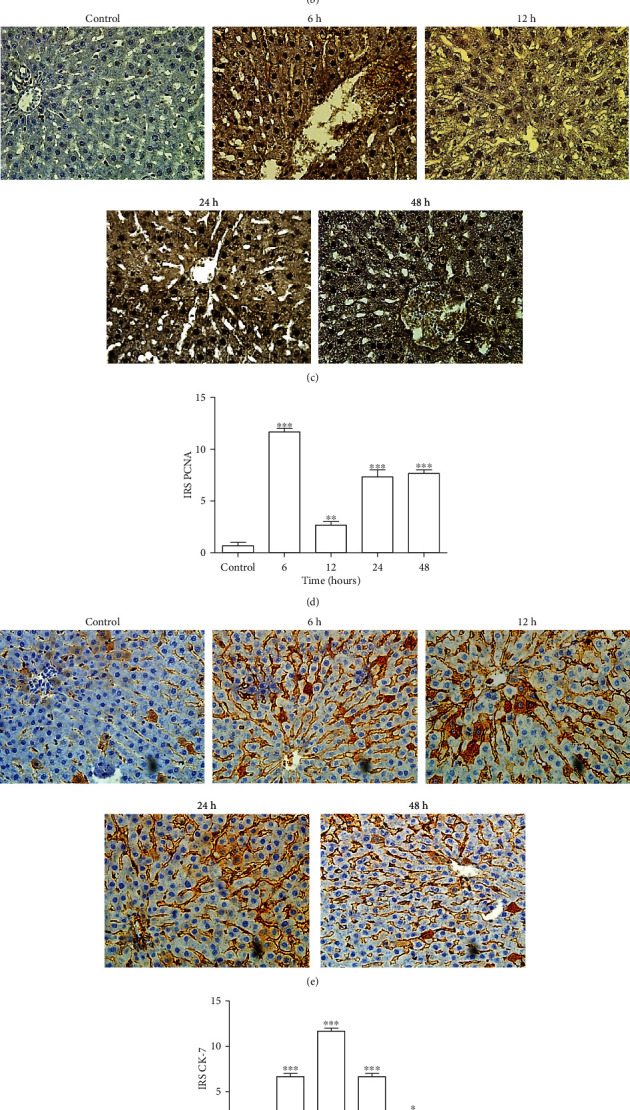
(a) Effect of short-term exposure of Tacrolimus on histopathological alterations in rat liver sections of control, 6, 12, 24, and 48 h treatment groups. PAS-D staining of liver control group showing intact liver architecture with almost no globules, while treatment groups indicate derangement of hepatocytes and presence of globules (arrows) due to collagen digestion (400x). Scale bar used is 50 *μ*m. (b) Effect of short-term exposure of Tacrolimus on histopathological alterations in rat liver sections of control, 6, 12, 24, and 48 h treatment groups. Reticulin staining of control group showing intact liver architecture with normal reticulin fibers, while treatment groups indicate derangement of hepatocytes and collapsed reticulin network (black gray color) (400x). Scale bar used is 50 *μ*m. (c) Effect of short-term exposure of Tacrolimus on PCNA immunostaining in rat liver tissue sections of control, 6, 12, 24, and 48 h treatment groups (400x). Scale bar used is 50 *μ*m. Degree of brown staining shows intensity of immune reaction. (d) IRS of PCNA in all experimental groups was scored and data was analysed using one-way ANOVA. Data is represented as mean ± SEM (*n* = 3). ^∗^*P* ≤ 0.05, ^∗∗^*P* ≤ 0.01, and ^∗∗∗^*P* ≤ 0.001. (e) Effect of short-term exposure of Tacrolimus on CK-7 immunostaining in rat liver tissue sections of control, 6, 12, 24, and 48 h treatment groups (400x). Scale bar used is 50 *μ*m. Degree of brown staining shows intensity of immune reaction. (f) IRS of CK-7 in all experimental groups was scored and data was analysed using one-way ANOVA. Data is represented as mean ± SEM (*n* = 3). ^∗^*P* ≤ 0.05, ^∗∗^*P* ≤ 0.01, ^∗∗∗^*P* ≤ 0.001. (g) Effect of short-term exposure of Tacrolimus on DNA damage in rat liver sections of control, 6, 12, 24, and 48 h treatment groups depicted by TUNEL assay staining (400x). Scale bar used is 50 *μ*m. (h) TUNEL-positive cells in all experimental groups were counted using ImageJ, and data was analysed using one-way ANOVA. Data is represented as mean ± SEM (*n* = 3). ^∗^*P* ≤ 0.05, ^∗∗^*P* ≤ 0.01, and ^∗∗∗^*P* ≤ 0.001.

Histopathological alterations from three fields, selected randomly, were analyzed by an independent pathologist to assess changes in liver architecture in all TAC-treated groups. PAS-D staining indicated intact liver architecture in the control but dilation of liver sinusoids; deranged hepatocyte with cytoplasmic swelling was evident as described previously [27] in all treatment groups. Furthermore, the presence of glycogen globules highlighted liver injury in all treatment groups (Figure 1(a)). The glycogen deposition was also confirmed in liver tissue homogenate.

Reticulin-stained slide section of the control group showed intact liver architecture with normal reticulin fibers, while treatment groups indicated loss of liver architecture due to collapse of reticulin fibers (Figure 1(b)). Condensed reticulin fibers appearing as grayish black fibers signal loss of underlying parenchyma and damage of tissue as hallmark of liver injury. Reticulin fibers are part of the extracellular matrix and give a clue of disturbed architecture of hepatic plates and collapse of reticulin framework [29].

### 3.2. Tacrolimus Exposure Results in Liver Damage and Enhanced Staining of PCNA and CK-7

Three fields were selected randomly, and slides were analyzed and evaluated by an independent pathologist. Stained slide sections were scored by immunoreactivity scoring (IRS) method [30]. Intensity of staining and percentage of immunopositive cells were determined to evaluate IRS. Furthermore, every tissue sample was classified into IRS points (0-12) as no, mild, moderate, and strong staining. A higher degree of brown staining of cytoplasm and nucleus normally shows a higher level of liver tissue damage and steatosis. Significant differences of immune reaction to PCNA and CK-7 antibodies were observed between experimental and control groups as shown in Figures 1(c) and 1(e), respectively. The tissue section used as a negative control did not show any background staining. As depicted by IRS, PCNA showed no immunoreactivity in control (0.67 ± 0.33) and strong immunoreactivity in the 6 h (12 ± 0.33), mild in the 12 h (2.7 ± 0.33), and moderate in the 24 h (7.3 ± 0.67) and 48 h (7.7 ± 0.33) treatment groups (Figure 1(d)). Likewise, in the case of CK-7, no immunostaining was observed in the control group (0.67 ± 0.33), moderate immunoreactivity in the 6 h (6.7 ± 0.33) and 24 h (6.7 ± 0.33), strong in the 12 h (12 ± 0.33), and mild in the 48 h (2.3 ± 0.33) treatment groups (Figure 1(f)).

### 3.3. Short-Term Tacrolimus Exposure Results in DNA Damage

Results indicated DNA damage due to TAC exposure in all treatment groups (Figure 1(g)). Three fields were selected randomly, and TUNEL-positive cells were counted by ImageJ software. Data revealed significant DNA damage in 6 (1772 ± 22.9), 12 (3885 ± 2.65), 24 (4857 ± 323), and 48 h (3312 ± 108) treatment groups as compared to the control group (59.3 ± 1.76). Maximum degree of DNA damage, however, was indicated by the 24 h treatment group (Figure 1(h)).

### 3.4. Increased Glycogen in Liver Tissue Homogenate due to TAC Exposure

**Figure 2:**
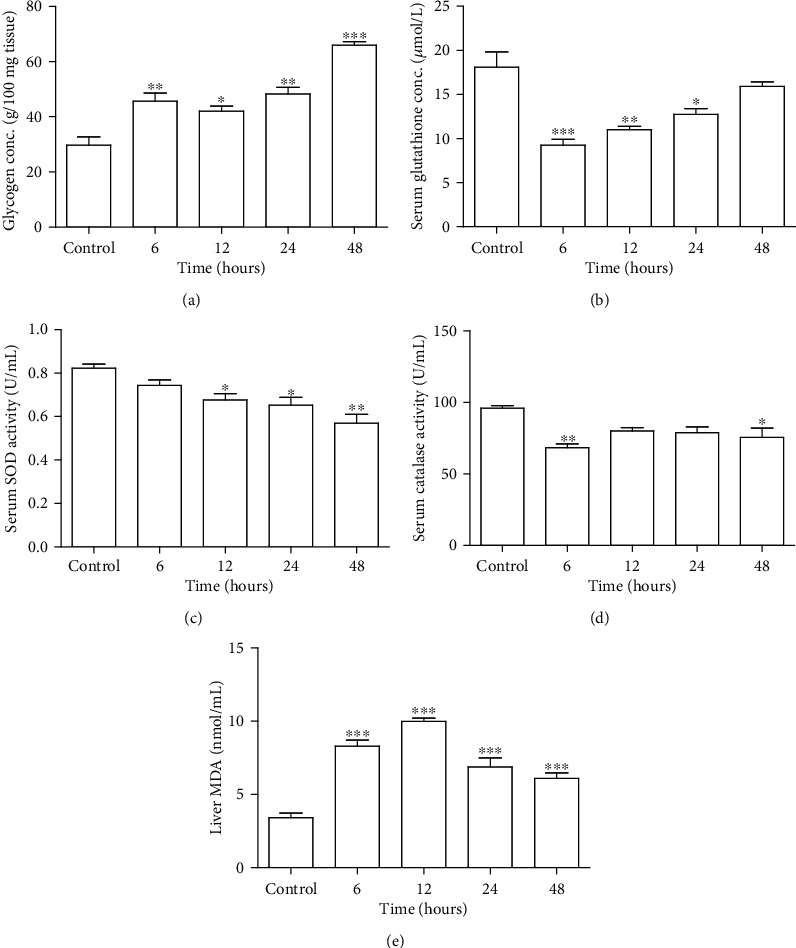
Effect of short-term Tacrolimus exposure on concentration of glycogen in liver tissue, serum antioxidants, and liver MDA level. (a) Increased glycogen in liver tissue homogenate is shown in graphical representation depicting the concentration measured in g/100 mg of tissue in control, 6-, 12-, 24-, and 48-hour treatment groups. Data is represented as mean ± SEM (*n* = 6). ^∗^*P* ≤ 0.05, ^∗∗^*P* ≤ 0.01, and ^∗∗∗^*P* ≤ 0.001. Serum antioxidant status and tissue MDA level are shown in graphical representation in control, 6-, 12-, 24-, and 48-hour treatment groups. (b) GSH concentration, (c) SOD activity, (d) CAT activity, and (e) MDA level. Data is represented as mean ± SEM (*n* = 6). ^∗^*P* ≤ 0.05, ^∗∗^*P* ≤ 0.01, and ^∗∗∗^*P* ≤ 0.001.

Concentration of glycogen in liver tissue homogenate was increased in 6 (46 ± 2.9 g/100 mg), 12 (42 ± 1.8 g/100 mg), 24 (48 ± 2.4 g/100 mg), and 48 h (66 ± 1.3 g/100 mg) treatment groups as compared to the control group (30 ± 2.9 g/100 mg) with maximum significant increase in the 48 h treatment group (Figure 2(a)).

### 3.5. Decrease in Serum Antioxidants due to Short-Term Exposure of Tacrolimus

#### 3.5.1. Glutathione Concentration

Concentration of serum GSH indicated a decreasing trend in all treatment groups as compared to the control group (Figure 2(b)). However, significant decrease was observed in the 6 (9.25 ± 0.66 *μ*mol/L), 12 (11.0 ± 0.38 *μ*mol/L), and 24 h (12.75 ± 0.62 *μ*mol/L) treatment group as compared to the control group (18.08 ± 1.74 *μ*mol/L) with no significant difference in the 48 h treatment group.

#### 3.5.2. Superoxide Dismutase Activity

Activity of serum SOD revealed a decline in all treatment groups as compared to the control, with a significantly reduced activity in the 12 (0.67 ± 0.02 U/mL), 24 (0.65 ± 0.03 U/mL), and 48 h (0.57 ± 0.04 U/mL) treatment groups as compared to the control group (0.82 ± 0.01 U/mL) (Figure 2(c)).

#### 3.5.3. Catalase Activity

Activity of catalase in serum samples was decreased in all treatment groups as compared to the control group (Figure 2(d)). However, significant decline in the CAT activity was only observed in the 6 (68 ± 2.7 U/mL) and 48 h (76 ± 11 U/mL) treatment groups as compared to the control group (96 ± 1.7 U/mL).

### 3.6. Lipid Peroxidation Assessment in Liver Revealed Increased MDA Level due to Short-Term Exposure of Tacrolimus

Lipid peroxidation status showed a significant increase in all treatment groups as compared to the control group. A markedly increased level of MDA was observed in the 6 (8.30 ± 0.41 nmol/mL), 12 (9.99 ± 0.22 nmol/mL), 24 (6.88 ± 0.62 nmol/mL), and 48 h (6.11 ± 0.35 nmol/mL) treatment groups as compared to the control group (3.42 ± 0.31 nmol/mL) (Figure 2(e)).

### 3.7. Tacrolimus Exposure Results in Upregulation of Cytokines

**Figure 3:**
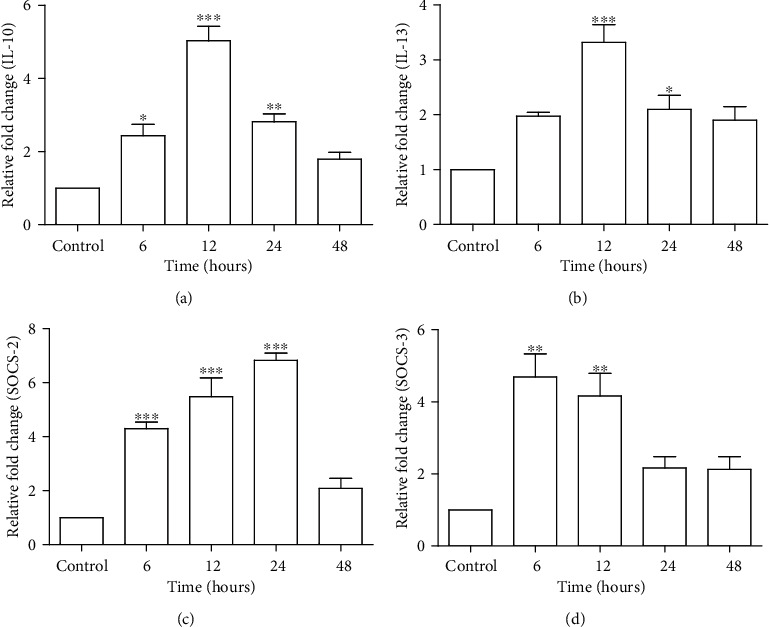
Effect of short-term Tacrolimus exposure on gene expression level of cytokines. RT-PCR results are shown in graphical representation depicting the relative fold change in control, 6-, 12-, 24-, and 48-hour treatment groups. (a) IL-10, (b) IL-13, (c) SOCS-2, and (d) SOCS-3. Data is represented as mean ± SEM (*n* = 6) and normalized with the expression of a housekeeping gene, GAPDH. ^∗^*P* ≤ 0.05, ^∗∗^*P* ≤ 0.01, and ^∗∗∗^*P* ≤ 0.001.

Gene expression analysis revealed an upregulation of IL-10, IL-13, SOCS-2, and SOCS-3 in experimental groups as compared to the control group as shown in Figure 3. IL-10 gene expression significantly increased in the 6 (2.43 ± 0.30-fold), 12 (5.03 ± 0.39-fold), and 24 h (2.81 ± 0.21-fold) treatment groups as compared to the control group (1.00 ± 0.00-fold) (Figure 3(a)), whereas the IL-13 gene expression showed a significant upregulation in the 12 (3.31 ± 0.06-fold) and 24 h (2.09 ± 0.25-fold) treatment groups only, as compared to the control group (1.00 ± 0.00-fold) (Figure 3(b)). In the case of SOCS-2 and SOCS-3, the gene expression profile of SOCS-2 significantly increased in the 6 h (4.30 ± 0.24-fold), 12 h (5.48 ± 0.69-fold), and 24 h (6.83 ± 0.26-fold) treatment groups as compared to the control group (1.00 ± 0.00-fold) (Figure 3(c)). And the expression pattern of SOCS-3 significantly increased only in the 6 h (4.69 ± 0.64-fold) and 12 h (4.16 ± 0.62-fold) treatment groups as compared to the control group (1.00 ± 0.00-fold) (Figure 3(d)).

## 4. Discussion

Although the use of immunosuppressive drugs has potentially lowered the risk of graft rejection, adverse effects in terms of prevalence of other diseases and life expectancy are still there [4]. Toxic effects of oral administration of TAC have also been described as liver inflammation coupled with activation of acute phase response (APR), with regulation of interferons and interleukins [26]. However, the short-term impact of TAC on oxidative stress has never been investigated. The current study was carried out to further explore the effects of short-term TAC exposure on antioxidant status and inflammation. The findings of this study revealed evident liver damage and glycogen deposition by significantly altering the liver architecture and change in antioxidant status of serum GSH and SOD, CAT, and liver lipid peroxidation, along with DNA damage, PCNA and CK-7 expression, and altered gene expression of cytokines.

Oxidative hepatic damage and change in liver architecture with significant decrease in GSH concentration, inhibition of SOD and CAT activity, and increased MDA level have been previously reported in a liver toxicity model of rats [31, 32], and a link between change in antioxidant status and liver histopathology owing to liver injury has been described [33]. Additionally, decreased activity of SOD and CAT, reduced GSH concentration, and increased MDA level have been suggested to be responsible for oxidative damage and apoptosis in TAC-induced diabetes mellitus in an animal model [34]. MDA reflects lipid peroxidation and plays a vital role on oxidative damage. These evidences support the notion that histopathological changes with decreased concentration of GSH, reduced activity of SOD and CAT, and increased level of MDA may be due to oxidative hepatic damage in experimental groups. Moreover, DNA damage in our study, as evidenced by TUNEL-positive cells in TAC-treated groups, might attribute to the decrease in antioxidant status and increased lipid peroxidation in the liver. Liver injury due to decreased GSH concentration, reduced SOD and CAT activity, elevated MDA level, and DNA damage has been described in previous studies due to the production of reactive oxygen species [35–37]. This fact suggests that oxidative stress can induce apoptosis in the liver, which in turn can further exacerbate inflammation.

Several factors, such as hypoxia, pathological injury, and cell proliferation via the NOD-like receptor inflammasome pathway, contribute to increased expression of PCNA and CK-7 in the liver [38–41]. Increased expression of CK-7 and PCNA has been reported in liver transplant patients due to recurrence of liver complications, drug toxicity-induced hypoxia, and liver fibrosis [42–44]. Increased expression of PCNA and CK-7 in current findings may imply liver injury associated with dysregulation of antioxidants and lipid peroxidation.

Cytokines IL-10 and IL-13 are considered as anti-inflammatory and upregulated in acute liver injury and liver fibrosis to prevent the damage [23, 45, 46]. Song et al. have reported that using sirolimus as an immunosuppressive drug resulted in increased level of IL-10 in liver transplant patients by affecting B cell regulatory cells [47]. Impaired activity of SOD and MDA level with upregulation of IL-10 and IL-13 expression indicate inflammation and acute liver injury due to production of ROS [45, 48, 49]. These cytokines are also responsible for activation of the suppressor of cytokine signaling (SOCS) genes which can activate other signal transduction pathways, such as the JACK/STAT pathway to promote cell proliferation and apoptosis [25]. A link between augmented expressions of SOCS-1, -2, and -3 in hepatocytes due to endotoxin-associated inflammation has been reported [50]. Previous studies have also confirmed an increased expression of SOCS-3, IL-10, and TNF*α* in liver steatosis patients [51]. As suggested in previous studies, upregulation of cytokines in current findings may also indicate an inflammatory response. Our results highlighted an augmented mRNA expression of IL-10, IL-13, SOCS-2, and SOCS-3, which may attribute to proinflammatory reaction mediated by change in antioxidant status and lipid peroxidation.

Taken together, this data implies that even short-term TAC exposure could induce histopathological changes in tandem with changes in serum antioxidant status, liver lipid peroxidation, DNA damage, overexpression of PCNA and CK-7, and an impact on hepatic gene expression of cytokines. Therefore, to minimize rejection-related issues, these factors should be considered in case of prolonged immunosuppressive therapy in order to improve life expectancy of transplant patients. However, further studies with a multifaceted approach should be conducted using combination drugs in a chronic model of TAC exposure on rats, which is a limitation of this study.

## Data Availability

Data supporting this research can be available upon request.
